# Optical recognition of constructs using hyperspectral imaging and detection (ORCHID)

**DOI:** 10.1038/s41598-022-25735-9

**Published:** 2022-12-07

**Authors:** Ren A. Odion, Tuan Vo-Dinh

**Affiliations:** 1 Fitzpatrick Institute for Photonics, Duke University, Durham, NC USA; 2 Department of Biomedical Engineering, Duke University, Durham, NC USA; 3 Department of Chemistry, Duke University, Durham, NC USA

**Keywords:** Biomedical engineering, Nanoparticles, Optical spectroscopy

## Abstract

Challenges to deep sample imaging have necessitated the development of special techniques such as spatially offset optical spectroscopy to collect signals that have travelled through several layers of tissue. However, these techniques provide only spectral information in one dimension (i.e., depth). Here, we describe a general and practical method, referred to as Optical Recognition of Constructs Using Hyperspectral Imaging and Detection (ORCHID). The sensing strategy integrates (1) the spatial offset detection concept by computationally binning 2D optical data associated with digital offsets based on selected radial pixel distances from the excitation source; (2) hyperspectral imaging using tunable filter; and (3) digital image binding and collation. ORCHID is a versatile modality that is designed to collect optical signals deep inside samples across three spatial (X, Y, Z) as well as spectral dimensions. The ORCHID method is applicable to various optical techniques that exhibit narrow-band structures, from Raman scattering to quantum dot luminescence. Samples containing surface-enhanced Raman scattering (SERS)-active gold nanostar probes and quantum dots embedded in gel were used to show a proof of principle for the ORCHID concept. The resulting hyperspectral data cube is shown to spatially locate target emitting nanoparticle volumes and provide spectral information for in-depth 3D imaging.

## Introduction

The capability to image targets in deep tissue is very important for many applications in the field of medicine. For instance, multiple imaging techniques are used for detecting tumors in vivo based on chemical and molecular differences that inherently manifest in the altered tumor environments of many cancers. Differences in oxygen consumption, energy generation, and density are among the number of changes in the tumor that key techniques such as Positron Emission Tomography (PET), Magnetic Resonance Imaging (MRI), and Computed Tomography (CT) aim to detect^1,2^. However, the widespread use of these imaging gold standards for cancer detection are ultimately limited by their high costs and requirement of skilled personnel for operation and interpretation. Alternative detection methods using optical techniques such as diffuse reflectance, fluorescence imaging, and Raman spectroscopy have been developed to detect the molecular changes in the tumor environment in a rapid and cheap manner by imparting light unto the tissue and detecting either the backscattered or re-emitted photons^3–5^. These optical techniques are highly sensitive to the chemical composition of tissue and have been shown to effectively discern the altered oxygenation and chemical balance of key molecules involved in energy production such NADH and FAD^4^. However, complex chemical compositions and tissue geometries may result in overlapped spectral signals in absorption and fluorescence, thus limiting the use of techniques such as fluorescence and diffuse reflectance, which rely on wide spectral signatures. Several optical techniques provide spectra with very narrow spectral structures such as Raman, quantum dot fluorescence, and rare earth luminescence.

Raman spectroscopy, which relies on the inelastic scattering of light, produces a very narrow-band spectral signature unique to the vibrational signatures of key specific chemical groups. This feature makes the Raman method a highly specific technique that can detect the unique spectral fingerprint of specific molecules of interest amidst non-target signals and interfering background signals. In the context of cancer detection, several studies have shown differences in the total spectral Raman differences in tumors due to varying amounts of lipid density. With a simple change in laser excitation wavelength, Raman spectroscopy can also be shifted into the near-IR spectral region (700–1100 nm), referred to as the “optical window”, where there is much less tissue absorption, allowing for greater tissue penetration^6^. However, the technique is largely limited by the intrinsically low Raman scattering cross-section of most molecules. Fortunately, the Raman signal can be greatly augmented with the use of metallic nanostructures that induce the phenomena of surface-enhanced Raman scattering (SERS).

Following the discovery of the SERS effect for highly polarizable small molecules, such as pyridine, benzoic acid and their derivatives, our laboratory first demonstrated the general applicability of SERS as an analytical technique that can be applied to a wide variety of chemicals including homocyclic and heterocyclic polyaromatic compounds^7–9^. For the following three decades, our team pioneered the development of SERS techniques and plasmonic nanoprobes for a wide variety of chemical analysis, biological monitoring, and medical applications^10–16^. Nanoparticles made of noble metal (silver, gold) exhibit enormous Raman signal enhancement due to the so-called “plasmonic” effect producing a strong electromagnetic (EM) field enhancement near the nanoparticle’s surface, producing the SERS effect, which has been shown to achieve single-molecule detection. By coupling a Raman active dye with a metallic nanoparticle with a unique geometry such as a gold nanostar (GNS), a very strong SERS enhancement can occur due to the “lightning rod” effect on the sharp tips of the nanoparticle^17–19^. We have developed this nanoparticle platform as a means of nucleic acid biomarker detection of several diseases as well as additional uses such as photothermal heat treatment of cancer^12,20–22^.

The SERS technique has been used for the detection of Raman-labeled GNS absorbed in tumors for in vivo cancer monitoring using a murine model has been reported^13,23,24^. These applications illustrate the selective detection of SERS signals only in one dimension. To overcome this limitation, the technique Spatially Offset Raman Spectroscopy (SORS) is used to selectively collect Raman scattered photons that have originated from deeper in the sample by creating a source to detector separation^25–27^. Building upon previous work coupling SORS and SERS for detecting targets through tissue, we have coupled the SERS-GNS nanoplatform with the Spatially Offset Raman Spectroscopy (SORS) technique as a means of detecting through highly scattering material such as bone tissue^28–32^. In this work we aim at developing a novel and general methodology that can be applied not only to Raman but also to other optical techniques exhibiting narrow-band spectral structures such as luminescence from quantum dots, rare earth species and upconverting nanomaterials.

For this reason, we refer to the proposed method as Spatially Offset of Narrowband Optical Spectroscopy (SONOS), which comprises SORS as well as other types of spatially offset luminescence spectroscopy (SOLS) from quantum dots, rare earth species and upconverting nanomaterials. It is important to note that a general offset detection concept is not new as it has also been used in Diffuse Optical Tomography and Fluorescence Tomography as a means of creating a depth resolved spatial map of the different chemical compositions^33–35^. However, SONOS has the advantage of producing much sharp spectral peaks that do not overlap, thus allowing for much higher multiplexing capabilities and higher signal to noise ratio. Building on previous works that detect the intrinsic Raman signals of key molecules, we utilize our SERS particles as means of detecting through the skull up to 50 mm deep and show the potential for detecting deeply seated brain cancer tumors once nanoparticles have selectively collected around the tissue^24,36^. However, to date the SORS method relies on a static optical configuration that relies on the light source being physically separated from the detector spot. The use of a fiber bundle with different radial collection fibers can effectively collect the different spatial offsets at once, but it requires the use of a custom-made fiber bundle and is effectively sampling only one spot at a time, providing information in one z-dimension (depth).

**Figure 1:**
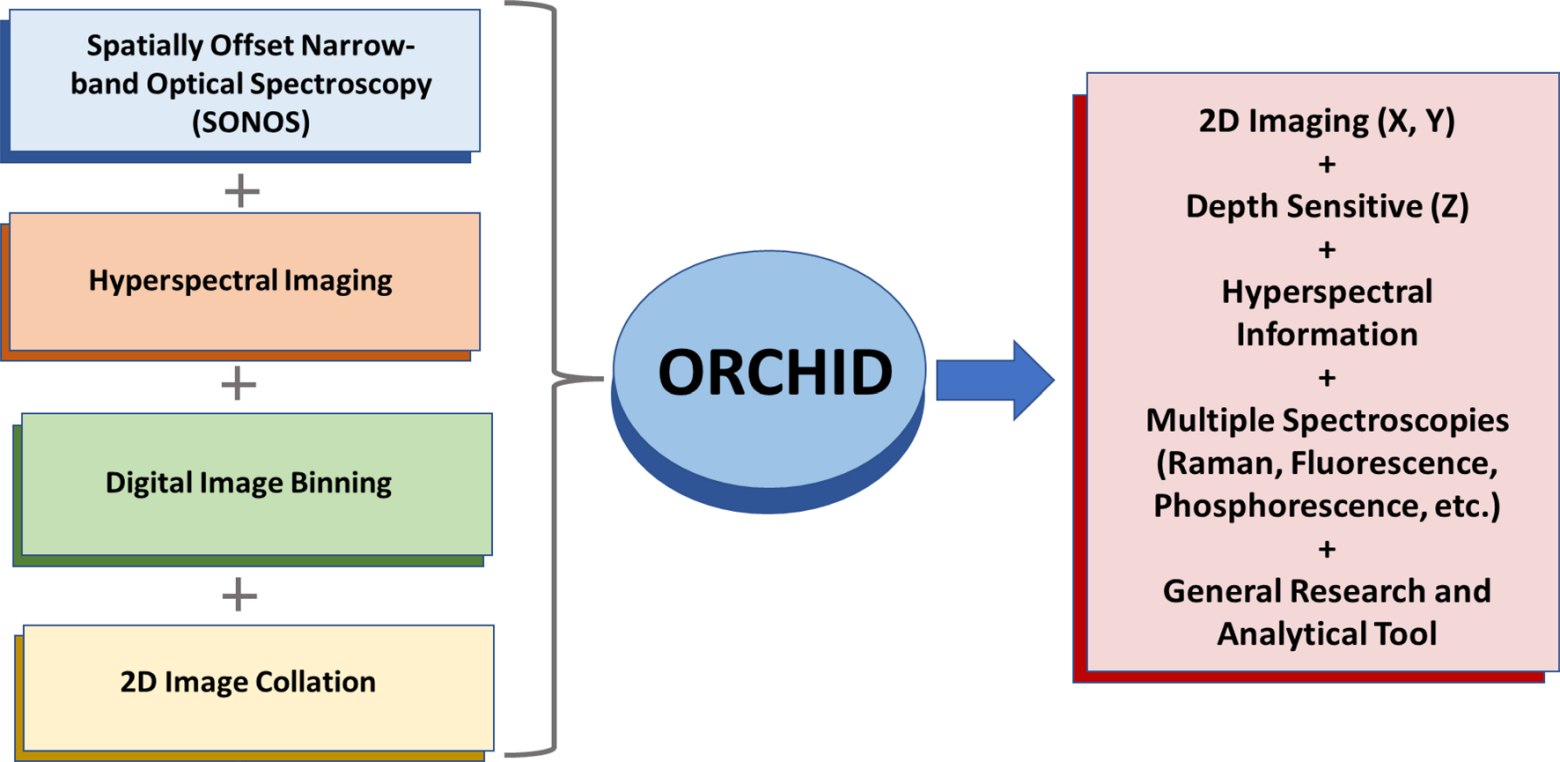
Schematic diagram of the ORCHID concept. The ORCHID system integrates several layers of instrumental systems and processing. Spatially offset methods consist of techniques that are used to selectively collect signal from deeper layers. Hyperspectral imaging integrates the spatial information collected from this method with spectral information to produce an information rich multidimensional dataset. Digital Image Binning and 2D Image Collation complete the system by integrating post processing techniques and stitching to produce a hyperspectral data cube that can be used to collocate signals with positions on the sample.

The new method, referred to as Optical Recognition of Constructs Using Hyperspectral Imaging and Detection (ORCHID) system is an integration of different techniques: (I) spatial offset detection concept, (II) hyperspectral imaging, (III) digital image binning, and (IV) 3D image collation (Fig. 1). ORCHID expands the spatial offset methodology into a 3D imaging modality in the XY plane and Z depth. This is done by combining a hyperspectral imaging system using a tunable filter, such as a spectrally scanned liquid crystal tunable filter (LCTF), and a two-dimensional charge coupled device (CCD). With ORCHID the recorded intensity data collected on the CCD are digitally binned within circular rings at different pixel radial distances from the excitation spot as a digital strategy for obtaining the different source to detector spatial offsets. By utilizing this concept, we can simultaneously collect the different depth-correlated signals from the offsets at once, and by spatially scanning the sample we are able to form a hyperspectral map of different spatial offsets. The result is a map of multiple hyperspectral data cubes that contain information on the spatial and spectral location of the SERS signal of the target. In this study, we demonstrate the ORCHID system’s capabilities by showing its ability to discern SERS signatures of different embedded GNS-dyes in agarose gel layers. We also show a mapping capability over a large field of view region of a gel to show the potential for hyperspectral imaging. The procedure represents a novel method for depth sensitive molecular imaging of tumor margins and provides a new development towards more accessible early cancer detection.

## Results

### The ORCHID digital offset concept

**Figure 2:**
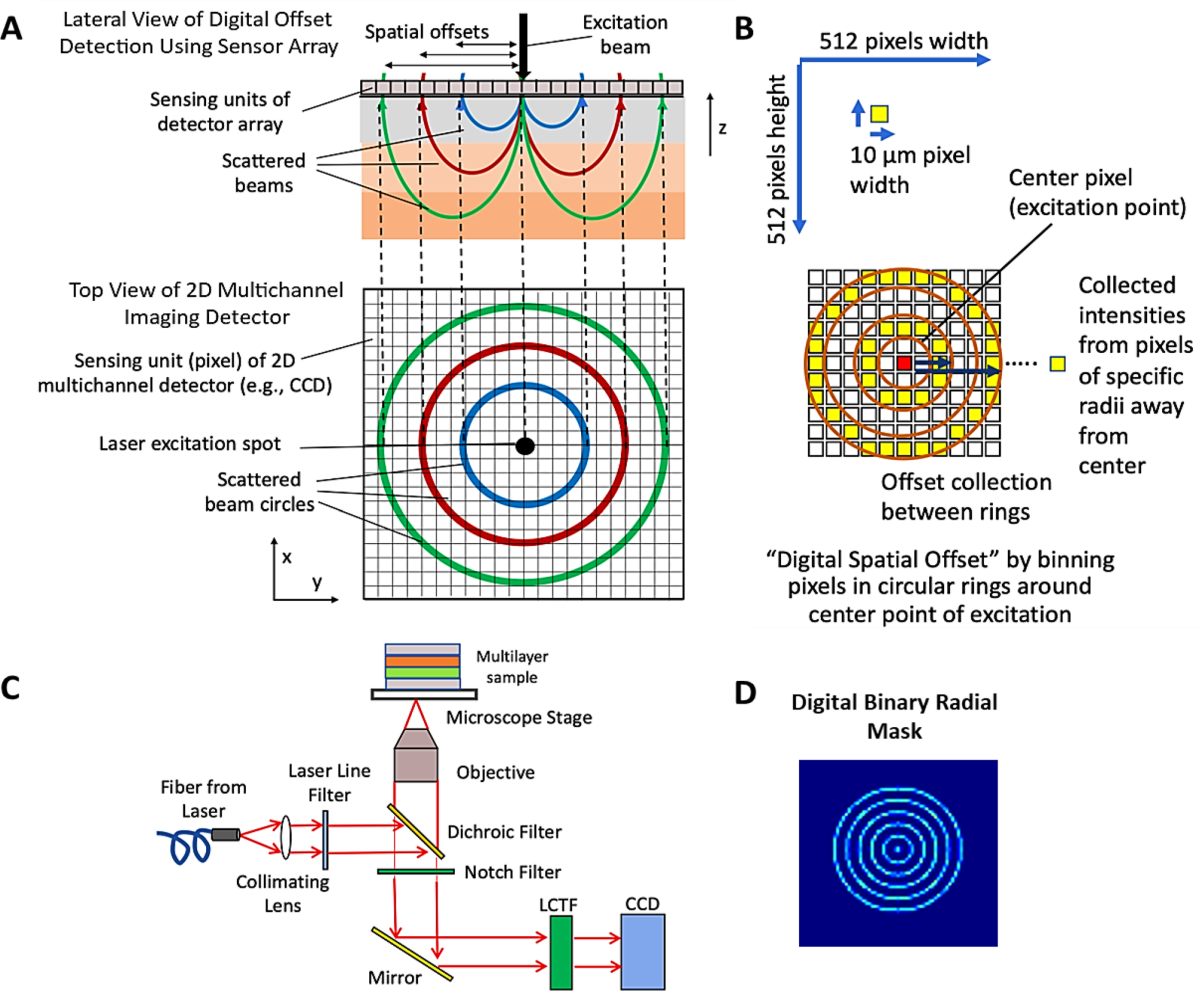
Optical configuration and spatial offset collection mechanism for the ORCHD system. (**A**) Lateral view of the collection of offset Raman signals. (**B**) Mapping of spatial offsets from a lateral slice to the imaging detector. (**C**) Schematic of ORCHID instrumental setup with a multilayer sample. (**D**) Binary radial mask used to collect spatially offset pixels from captured CCD image. Each ring in the mask isolates a collection of pixels representing signals of a specific spatial offset. The pixels are subsequently summed to produce an intensity value that will part of the spectral component of ORCHID.”

The spatially offset method of SONOS, which can be applied to Raman and luminescence, is fundamentally reliant on the calibration of spectra different source-to-detector separations. This spectral calibration process is most reliable with spectra exhibiting narrowband emission. Spatial offsets in most SONOS systems physically separate the focal excitation spot from the collection spot in specific distances. Several configurations have been previously used for Raman in SORS. However, the spatially offset approach can also be applied to luminescence techniques that exhibit narrow-band emission spectra such as luminescence from various species such as quantum dots, rare-earth species, and upconverting particles. The spatially offset strategy can be achieved using two lenses whose focal spots are physically separated, while other configurations using a ringed excitation of specific radial amounts can serve as the offset. An optical fiber bundle may also be utilized in similar manner wherein the excitation and collection fibers can be physically separated from each other (Fig. 2A). Here, the ORCHID system does not require a fiber bundle with different radial collection fibers to achieve the offset separating mechanism; the ORCHID system relies instead on computationally separated pixels that are at specific radial distances from the center of the two-dimensional CCD detector (Fig. 2A). The CCD with a pixel width of 12 µm and a total of 512 × 512 pixels can be radially binned at specific distances from the center by computationally selecting pixels a set distances, representing the spatial offsets of SONOS (Fig. 2B).

### Optical configuration of ORCHID system

**Figure 3:**
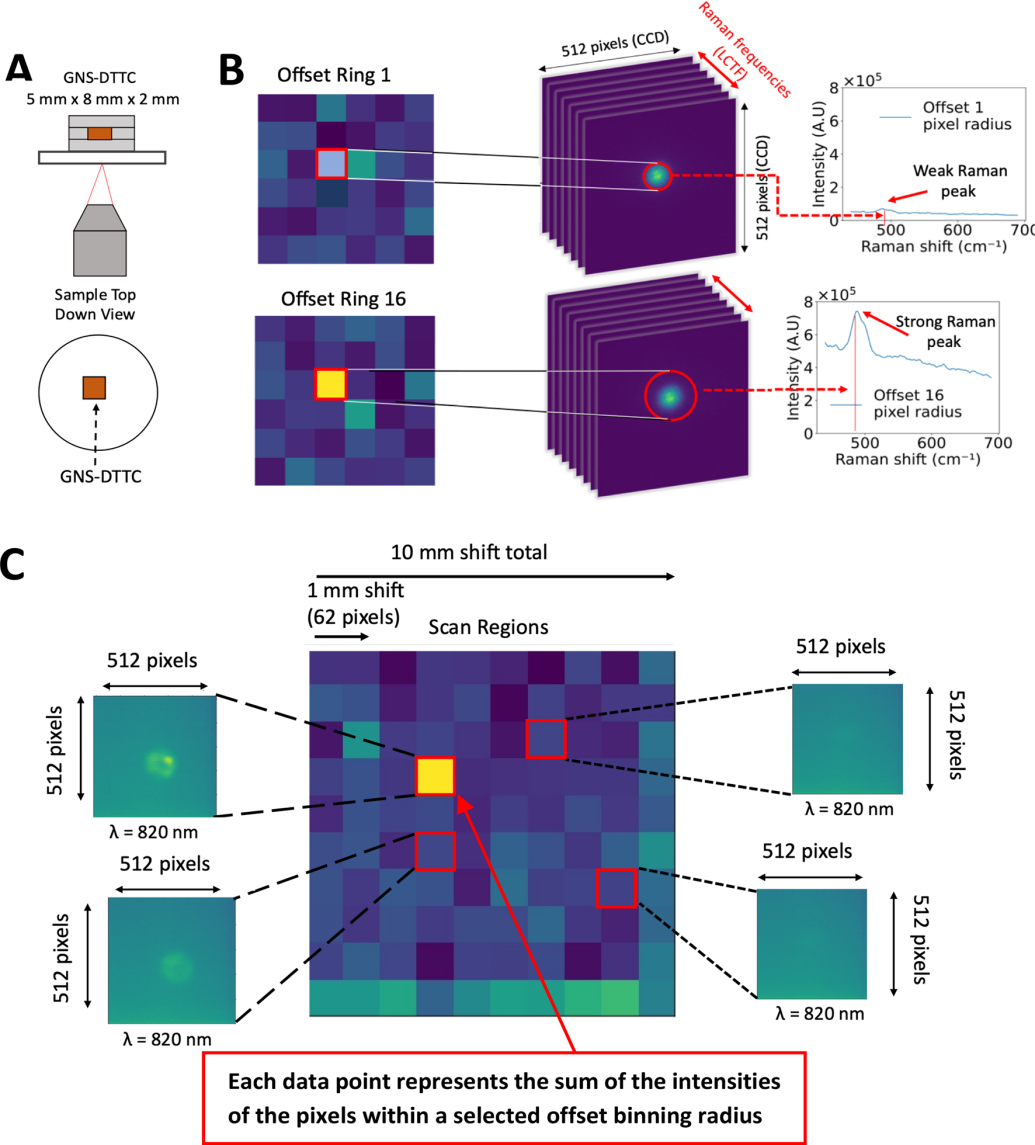
Demonstration of embedded single Raman layer detection with the ORCHID system. (**A**) Embedded GNS-DTTC gel sample of 2 mm thickness. (**B**) Hyperspectral imaging map of sample showing high Raman signal collection with greater spatial offset. The hyperspectral cube data is processed across different frequencies and subsequently binned and summed to produce the Raman spectra. The radially binned pixels are summed as a total intensity corresponding to a scanned location. The effect of larger radial binning shows a higher contrast for the GNS-DTTC section of the gel. (**C**) A larger spatial map showing the individual collected images at the corresponding Raman peak frequency (497 cm^−1^). The central image represents the total scanned images collected together where each data point on the image represents the sum of the spatially offset CCD image pixels corresponding to a selected binning radius. The brighter data points in the 2D image correspond to higher intensity values of GNS-DTTC gel.

**Figure 4:**
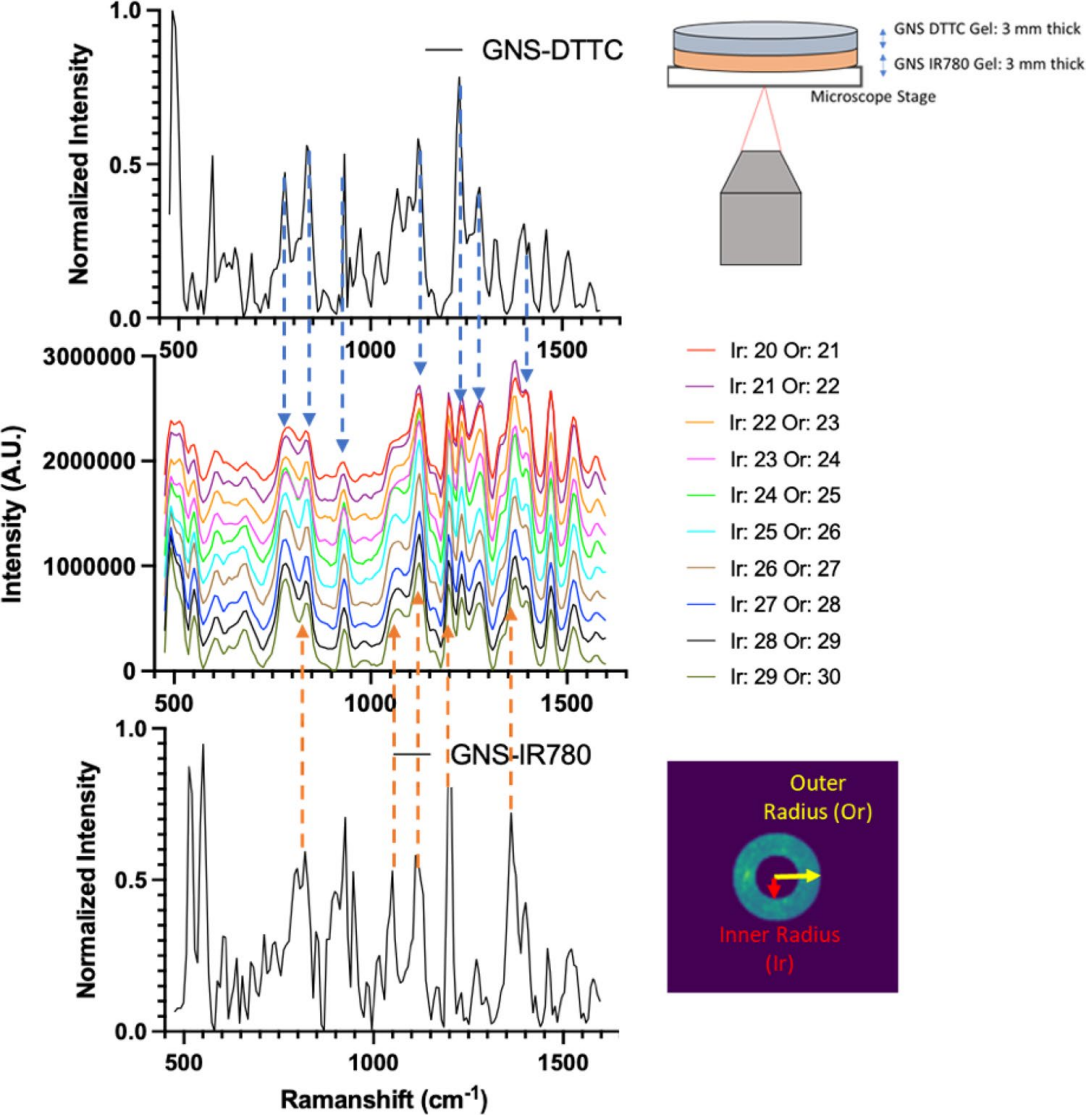
Detection of Raman from a dual layer sample. Two 3 mm thick gels containing GNS-DTTC and GNS-IR780 are stacked and probed with the ORCHID system at different spectral frequencies. The Raman spectra of pure GNS-dye are shown above and below a waterfall plot of different collected spectra at different radial binnings. The plot labels Ir (Inner radius) and Or (Outer radius) are the spatial offset binning. At greater spatial binning radii, spectral peaks coming from the farther layer become more prominent as shown with the changing Raman spectral peak intensities changing from each respective GNS-dye.

The ORCHID optical system shown in Fig. 2C comprises of an inverted optical microscope setup (Olympus Corporation, Shinjuku, Tokyo, Japan) coupled with a 785-nm laser that is collimated and sent to a dichroic mirror configuration where it is focused with a 10 × objective (Thorlabs, New Jersey, USA) towards a sample seated on a motorized linear stage (Zaber Technologies Inc., Vancouver, British Columbia, Canada). The backscattered signal is collected and passed through a Notch filter before going through a spectrally scanned dual-LCTF system mounted in front of a two-dimensional (512 × 512 pixels) CCD array (Princeton Instruments, New Jersey, USA). The LCTFs are programmatically scanned at selected spectral increments (e.g., 1 nm) and at a selected exposure time (e.g., 1 s). The sample is then spatially scanned in the XY plane using the motorized stage at selected increments (5 mm or less) to produce a spatial map that can localize the placement of small gel targets of 5 mm width within a larger gel volume. The resolution in the XY plane may reach optical resolutions of about 1 µm with smaller spatial increments, while Z axis resolution can be several microns depending on the sample size and the desired spatial resolution. Figure 2D shows the binary radial mask used to bin the CCD image data to isolate the pixels of specific spatial offset using different radial rings. These spatially offset pixels are later used to produce spatially offset maps and spectra in Figs. 3 and 4 respectively.

### ORCHID XY spatial image reconstruction

We first demonstrate the capability of the ORCHID system to produce a hyperspectral spatial map of a sample system, which consists of an agarose gel sample with an embedded layer containing gold nanostars linked with a DTTC Raman Dye (GNS-DTTC) in the middle (Fig. 3A). The GNS-DTTC is a small 5 mm by 5 mm cube sandwiched between 5 mm thick gel layers. Our previous study has shown that SERS-labeled GNS injected into a mouse model are preferentially absorbed into tumors due to the Enhanced Permeability and Retention (EPR) effect of tumor vasculature^16,23,37^. The EPR effect is a result of the inherent leakiness of the tumor vasculature which is often underdeveloped and allows nanoparticles to escape circulation and accumulate passively in tumors. Also, the retention of nanoparticles in the tumor is enhanced by the lack of an efficient lymphatic system which would normally carry extravasated fluid back into circulation. In this study, the agarose gel simulates a tissue sample and the GNS-DTTC cube represents a tumor that has absorbed GNS nanoprobes. The ORCHID system is scanned across this sample spatially and spectrally as means of spatially localizing the XY position of the GNS-DTTC cube. The stage is moved at increments of 1 mm and the LCTF wavelength was scanned at increments of 1 nm from 800 to 830 nm. The exposure was set to 1 s and selected to capture the data at each combination of spatial and spectral position. A slice at 820 nm of the stitched hyperspectral data cube is shown where the increased yellow intensity on the map shows the position of the GNS-DTTC volume position whose SERS spectra has a local peak at that wavelength (Fig. 3B). The resulting spectra from each pixel binning was obtained by collecting the imaging pixels at specific spatial offset (pixel radii) and summing across the different frequencies (Fig. 3C).

### ORCHID digital radial binning offset for in depth sensing

We used a simplified dual-layer gel sample of GNS-DTTC and GNS-IR780 that was stacked to illustrate the digital radial binning concept for in depth sensing. Each layer was 3-mm thick where the latter occupied the bottom layer, and the former occupied the top layer (Fig. 4). Hyperspectral data were collected at a single spot, where the LCTF was scanned from 800 to 880 nm. The resulting image arrays were computationally masked at different radial distances, representing spatial offsets by the distance of multiple 12 µm pixels as shown in Fig. 2B. A “waterfall” collection of different spatial offset spectra shows the change in spectral shape across increasing spatial offsets. Above and below this waterfall plot are the reference GNS-dye spectra with arrows pointing the unique peaks in each dye. In particular, the prominence of SERS peak belonging to one or the other GNS-dye changes indicating the ability of the system to discern between layers of the SERS targets of at least 3 mm. With greater spatial offset radial binning distances, the Raman peaks of the farther layer become more apparent, demonstrating that recovery of the Raman spectra from the deeply embedded layer is possible.

### ORCHID mapping and detection of embedded dual layer GNS-dye gels

**Figure 5:**
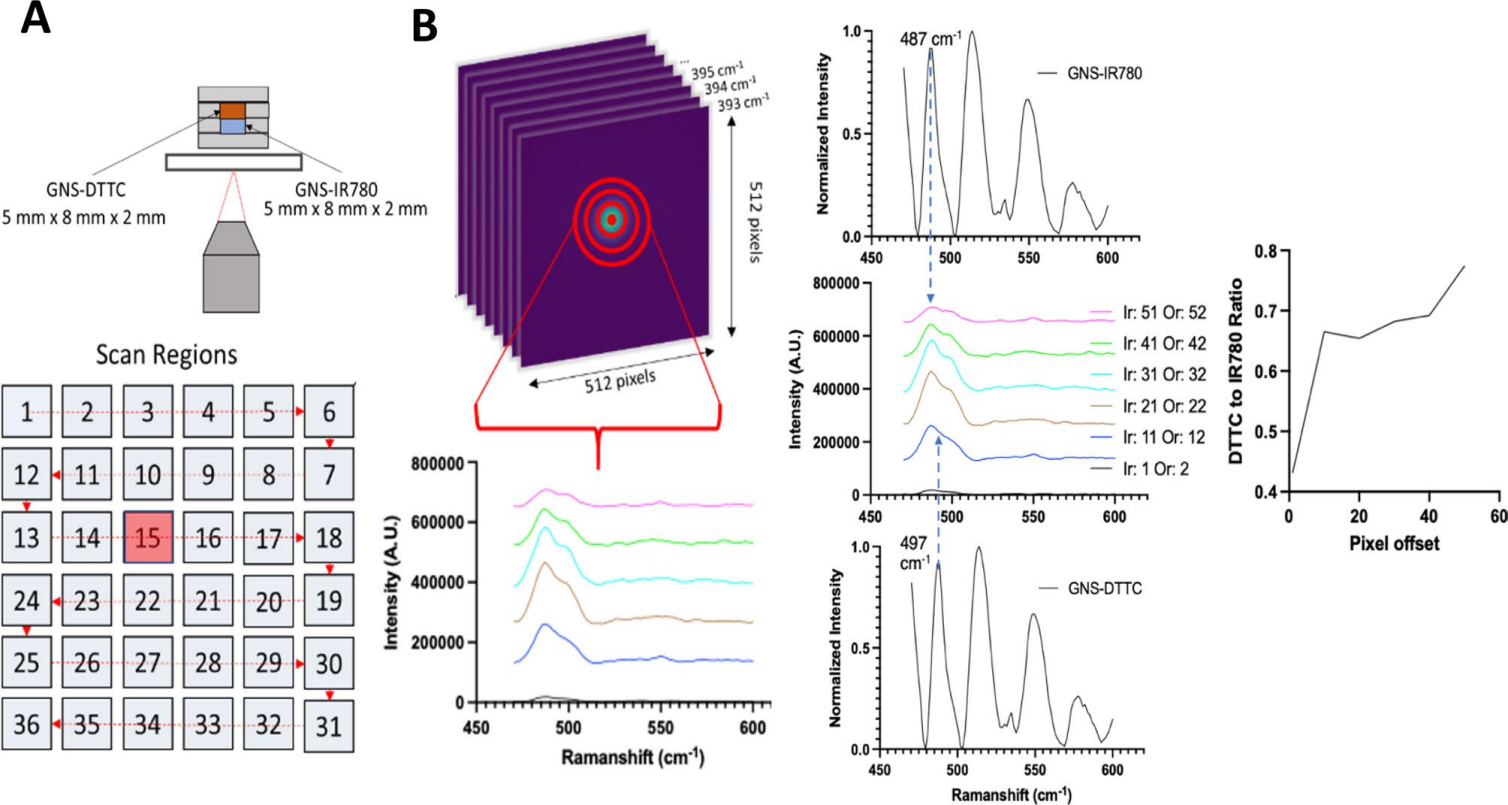
Detection of Raman from a dual layer sample. (**A**) Two 2 mm thick gels containing GNS-DTTC and GNS-IR780 are stacked and embedded between 2 mm thick gel layers and subsequently probed with the ORCHID system at different spectral frequencies. The resulting Raman spectra are obtained in the highlighted region containing the stacked GNS-dye sample. (**B**) As an example, the hyperspectral data cube at position 15 (highlighted in red) in the scan region is displayed with corresponding radial binnings that produce different spectra. This position represents the spatial location of the SERS-GNS gel volumes within the greater gel sample. A waterfall plot comparing the different inner and outer radial offsets is shown with the respective reference spectra of each dye above and below. As before, increasing spatial offsets resulted in the further out GNS-DTTC signal to become more prominent. Ir and Or stands for inner radius and outer radius respectively and represent the spatial offset disks.”

To demonstrate the ORCHID mapping capability, we used an embedded dual layer sample sandwiched between 5 mm thick gel layers (Fig. 5A). The XY plane mapping is finally combined with the binned radial spatial offsets (shown as disks of width 1 pixel centrally spaced around the excitation point). Supplementary Fig. 1 details the image array and binary mask used to spatially bin the image data. Data were collected in a similar manner as the previous experiments but now the data are formed together to make a hyperspectral data map (Fig. 5B). With the spectral peaks of each GNS-dye identified, a ratio-metric graph of between the dye peaks of GNS-DTTC and GNS-IR780 are depicted to show the spectral change as the spatial offsets are increased (Fig. 5B). Additional spatial binning configurations are detailed in Supplementary Fig. 2. The data ultimately shows the prevalence of the farther layer GNS-dye with greater offset in agreement with the ORCHID concept.

### Demonstration of imaging on quantum dots infused gel samples

**Figure 6:**
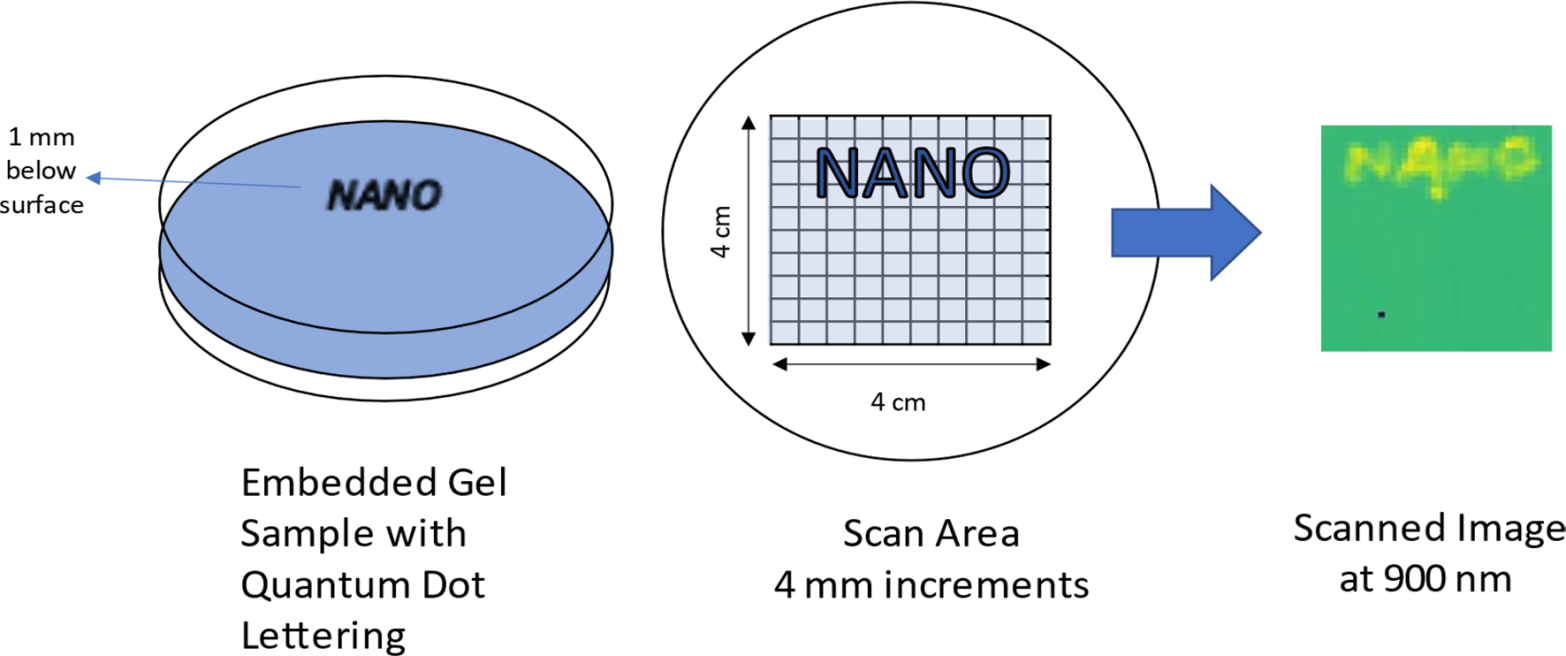
Embedded quantum dot gel sample and scanning scheme for demonstrating the ability to resolve at millimeter scale. The scanned image shown here is the slice collected at 900 nm and the CCD images at each scan position are summed without radial binning at each point to create the resulting image on the right.

To show the ability of the ORCHID system to resolve objects in the XY plane, we created a gel sample infused with quantum dots in the form of the word “NANO” about 1 mm below the surface of the gel. Figure 6 shows the sample, which was contained in a petri dish and raster scanned across a 4 cm × 4 cm area at 4-mm stage movement increments while data were collected at 100-ms exposure time. In this example, the resulting image shows that the system can resolve millimeter scale features such as the letters.

**Figure 7:**
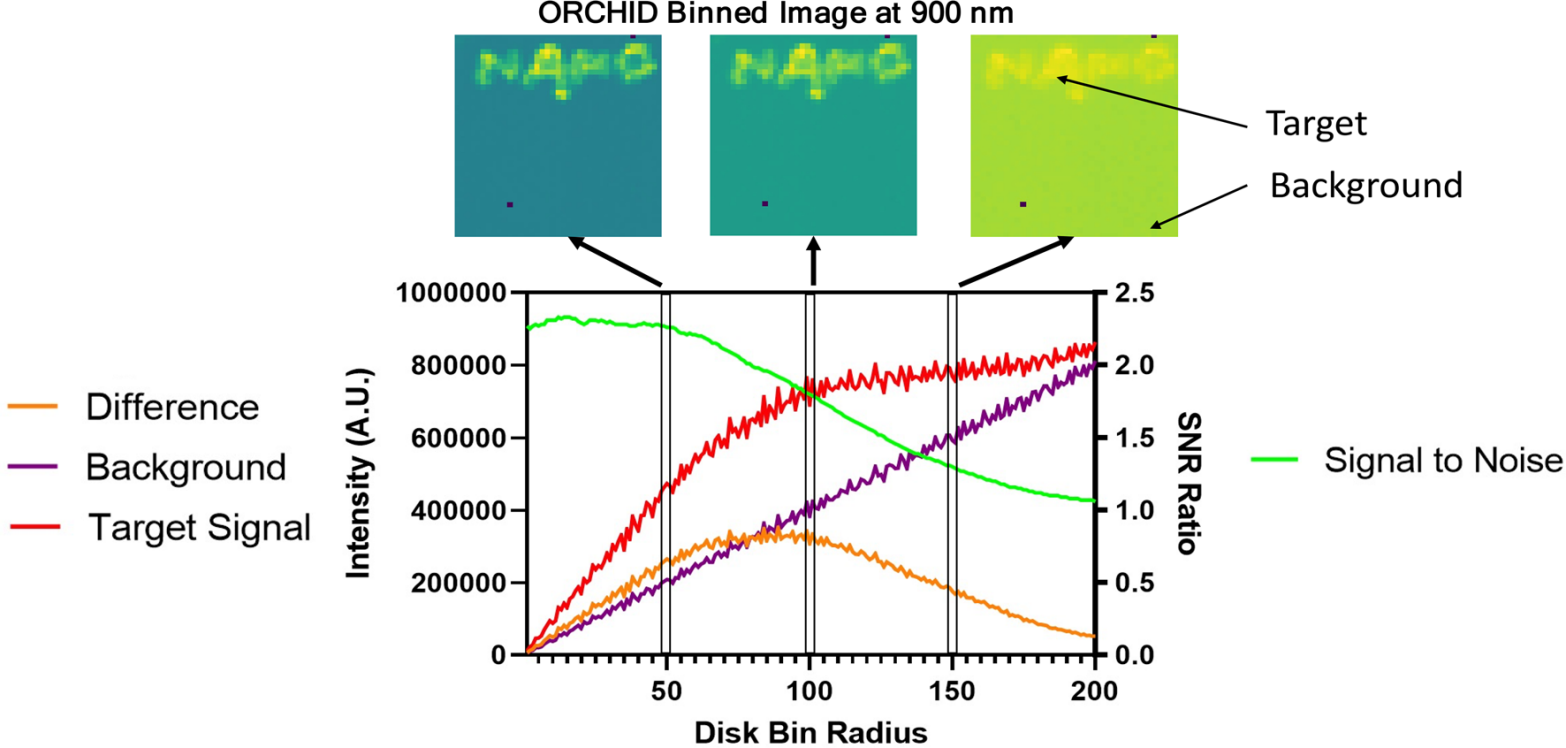
Plotted comparisons of target signal pixel (inside the “NANO”) and a background pixel as a function of disk binning radius (of 1 pixel width) used to form the raw images. Three resultant images are produced at 50, 100, and 150 disk bin radii each showing a changing contrast in each image. With increasing bin radius, the target signal difference from background is greatest between 50 and 100 pixel disk bin radius. This results in the greatest contrast in the middle resultant binned image shown above.

The images obtained are binned separately using different varying disk radii (see Supplementary Fig. 1). A pixel region where the target signal is located (within the “NANO” word) is plotted in Fig. 7 against this bin radii to show the effect of the peak radial bin on signal collection parameters: target signal, background signal, difference absolute signal (i.e., background-subtracted target). The target signal is the photon count collected at the location of quantum dots, for example at the letter “A” as indicated in Fig. 7. The background signal is the photon count collected at an area without quantum dots (i.e., outside the word “NANO”). The background signal, represented as the purple line, increases linearly with the bin radius as expected since a larger bin radius corresponds to a larger circle of a binned detection area as shown in Fig. 2B. On the other hand, the target signal, represented as the red curve, initially increases with increasing bin radius, but the rate of increase diminishes after the 75-pixel radii to level off. As the result, the absolute signal (i.e., background subtracted signal), represented as the orange curve, reaches a maximum at around 75-pixel radii. The signal-to-noise ratio is also plotted on the right axis showing the decrease in signal after 50 pixels of radial binning. The corresponding image at three radial binning configurations are shown above the plot to illustrate the differences in image quality at those respective binning settings.

## Discussion

Spatially Offset Raman spectroscopy is most crucially used to probe the molecular composition of a sample that is especially thick and turbid. The concept of source and detector separation has been implemented in a variety of ways, most commonly with a physical separation between the excitation and collection points in order to provide depth-related spectral information for one dimension (Z) The ORCHID system leverages the multichannel imaging approach of a CCD where spatial offsets are directly mapped and digitally binned in selected regions on the 2D (XY) imaging array detector in order to produce 3D data (XYZ) with depth information.

Furthermore, the use of liquid crystal tunable filters leverages the hyperspectral imaging approach where each image can be scanned spectrally through multiple wavelengths, thus producing a four-dimensional (4D) data hypercube (XYZ, wavelength). The ORCHID method utilizes a simple configuration that leverages the unique advantages of an LCTF. With this solid-state tunable filter device, we can scan across a sample obtain a hyperspectral cube map containing data in 3 axial directions and 1 frequency direction. We demonstrate the ability to achieve SORS at single points and SORS at points in a map up to 100 mm^2^ area. These capabilities will allow of much larger sample imaging and ultimately provide molecular information in different dimensions. The ORCHID system can currently obtain signal up to half a cm, but this Z resolution is highly dependent on the type of sample, level of scattering, and quality of the detector. SORS has a theoretical resolution in the order of centimeters and can feasibly be achieved with the ORCHID system with further optimization to the optical setup.

Raman signal in the lower layers notably becomes more prominent with spatial offset, signifying the selective ability of the ORCHID system on the Z axis direction. It is important to note that signal shape and intensity are highly variable and attempts of a calibration in a simple gel phantom results in a model that is often not transferrable to more complex samples containing non-Raman signal and background noise or those that have an irregular shape with different boundaries between layers. Agarose gel phantoms are idealized media often having low scattering and absorption in the near IR range, thus allowing for the SERS signal of GNS to be the primary signal source. However, real tissue is highly complex and has optical properties (absorption and scattering) which could affect excitation laser light penetration and target signal propagation. Additionally, there are also interfering background sources such as intrinsic fluorescence arising from biological tissue components that can potentially interfere with the target signal. This is a large hurdle in applications of SORS *in-vivo* and typically limits its usefulness to samples that already have a strong intrinsic Raman scattering property such as bone^30,32,38,39^. The ORCHID system offers a way to circumvent this issue by taking advantage of emission signals of particles such as SERS gold nanostars and quantum dots that exhibit sharp and identifiable spectral peaks. Additionally, the unique scanning and binning imaging process can be used to localize signals to areas that specifically aggregate particles. In cancer diagnostics, this approach can be used for detecting tumors by taking advantage of the well-known enhanced permeability and retention feature, which involves the natural propensity of nanoparticles to extravasate from the tumor vascular network and accumulate in and around cancer cells^16,23,37^. In this study, we have used gel phantoms whereby the GNS-loaded volume corresponds to tumor because our previous studies have demonstrated that injected GNS to mouse models can selectively accumulate into tumors due to this EPR effect. To that end, we showed that the ORCHID system can create a hyperspectral SERS dataset in multiple spatial dimensions, providing information that could help distinguish tumor tissue from non-tumor using gel phantoms.

Acquiring a high-resolution multidimensional hyperspectral data is time consuming due to the need to scan across a sample, and thus an optimization in the scanning methodology must be considered. In this proof of principle demonstration, we selected a stage increment of 5 mm. Higher spatial resolution would involve smaller stage increment, thus high number of measurement data points and therefore longer data acquisition time. Tradeoffs between data collection time and data volume must be made based on the needs of the applications. The ORCHID system has a theoretical resolution set at the optical resolution limit dictated by the objective lens and the laser wavelength used. At near-IR wavelengths, this can be around 0.7 to 1 µm range as for the typical epi-illumination microscope in the XY plane. However, mechanical limitations to the physical scanning of the stage itself could slow collection time, and for this initial configuration we utilized a standard microscope stage that has a typical movement speed of around 200 mm/s. Faster scanning can be achieved by using high speed stages or alternatively galvanic mirrors, both of which can dramatically decrease the data collection time by orders of magnitude.

Signal quantification requires more experimental tuning as the different spatial offsets have different signal-to-noise ratios (SNRs) since larger spatial offsets contain more pixels in a larger circle to receive the outgoing Raman/luminescence photons. In Fig. 7. we show that there is a maximum radial offset of around 75 to 80 pixels that maximizes the signal corresponding to the depth of the target (1 mm below the surface). This demonstrates the ability to correlate depth to the ORCHID signal collected. Corresponding images at 50, 100, and 150 binned radial pixels show correlation with the trend of the signal ratio in the plotted graph peaking at around 80 pixels. However, an important factor to consider is that the SNR gradually decreases in the larger radial binning due to the signal becoming more spread out along the binned pixels. It is important to note that this technique creates a tradeoff in maximal depth and depth resolution. Our previous work on using SORS through an animal skull has allowed for detection of signal up to 50 mm deep, but the current ORCHID system has so far been demonstrated to resolve down to 15 mm in this work^32^. This depth constraint could be addressed with the use of larger radial binning (i.e. spatial offset) values which would produce signals for increased depth. In fact, Fig. 7. indicates that there is a decreasing difference between target signal to background with greater spatial offsets, ultimately limiting the maximal depth potential of the ORCHID system. One way to increase this signal to background difference and maximal depth detection is to increase the radial binning thickness. It is noteworthy that there is a tradeoff between depth resolution and radial binning thickness due to mixing of signals along the layers as more of the axial positions are sampled.

The ORCHID system as demonstrated in this work shows a potential for creating imaging maps with molecular specificity due to its ability to obtain multi-dimensional hyperspectral data. In particular, deep sample imaging such as for tumors containing SERS active GNS is possible as the modality allows for spatially sensitive detection of spectrally unique signal. Molecular tissue margining for live tumor resection is an application of ORCHID that will greatly augment existing surgical cancer treatments which traditionally rely on offline imaging modalities such as CT or MRI. ORCHID also has the benefit of probing through depth using image processing alone unlike existing fiber bundles that have set source-detector separation configurations. The current ORCHID system is a prototype device designed to provide a proof of principle demonstration. Future development could further improve the system. Namely, the collection speed of the ORCHID system could be designed to be faster. To address this issue, galvanic mirrors for beam steering can be used in place of scanning for much greater scanning speeds.

Another limitation of ORCHID and diffuse optical modalities in general is spectral mixing, in which signals from other diffuse sources (e.g. interfering autofluorescence from tissue media) in the sample are mixed with the target signal. Numerous attempts have been made to separate the spectral signals in terms of layers based on signal intensity and a-priori knowledge of the top layer spectra^32,40^. Multivariate statistical techniques in particular use features of the spectra to unmix the signals to map towards the layers, but these methods typically require relatively clean data and will be negatively impacted by non-Raman features in the spectra^40,41^. The field of chemometrics leveraged such classical statistical techniques for spectral decomposition, but recently there has been a re-emerging trend towards using deep learning neural networks for data analysis and prediction^42–45^. Thus, machine learning technology can be leveraged to better decompose the different signals in the target. Imaging in particular has seen explosive development in using convolutional neural networks for feature extraction and labelling, and such methods are now being utilized in hyperspectral imaging^43,46,47^. Future integration with neural networks can be used to solve the inverse problem of having to know what type of and where the signal came from based on the detected signal in a system like ORCHID.

## Conclusion

The ORCHID system represents a novel modality for probing deeply embedded targets in highly scattering tissue. This has significant impact on its use as a cancer detection mechanism for in vivo tissue sensing as current modalities (e.g., PET, MRI, CT) are prohibitively expensive or comes with trade-offs such as the lack of molecular information. The ORCHID method is suitable for use with optical techniques exhibiting narrow-band structures, such as Raman scattering, luminescence from quantum dots, rare earth species, and upconverting nanomaterials. ORCHID combines the strengths of the digital spatial offset concept, hyperspectral imaging, and digital image binding and collation to create a dataset containing information ranging from spatial distribution to molecular makeup. For instance, the ORCHID system can provide a real-time monitoring and detection system cancer margin detection when used to detect gold nanostars that accumulate preferentially in tumors due to the EPR effect^16,23,37^. Applying the ORCHID system with SERS gene nanoprobes would provide a potential future in which gene expression can be monitored in-vivo in tissue or plant systems^12,23,48^. In conclusion, the ORCHID system integrates hyperspectral imaging with digital data processing in order to provide 3D spatial and spectral information for various spectroscopic techniques including Raman, fluorescence, and phosphorescence.

## Methods

### ORCHID instrumentation

The ORCHID optical instrumentation is shown in Fig. 2C. The system comprises of an inverted optical microscope setup (Olympus Corporation, Shinjuku, Tokyo, Japan) coupled to a 785-nm laser. The laser source is then collimated through dichroic mirror configuration and focused with a 10 × objective (Thorlabs, New Jersey, USA) to a sample seated on a motorized linear stage (Zaber Technologies Inc., Vancouver, British Columbia, Canada). The signal is subsequently collected through a Notch filter and then through a spectrally scanned dual-LCTF system mounted in front of a two-dimensional (512 × 512 pixels) CCD camera (Princeton Instruments, New Jersey, USA). The LCTFs are set to filter at 1 nm spectral increments and the camera acquisition is set to a value 1 s exposure time. The sample is then scanned in the XY plane using the motorized stage at 5 mm increments to produce a spatial map.

### ORCHID system processing

The system is programmed using the Python programming language (Version 3.7.5) with direct device interfacing for the camera, stage, and filters. Data treatment and analysis for forming the images and spectra were done with Python using the Numpy and Matplotlib libraries. Finalized graphs were created using GraphPad Prism. The spatial offset data were created by multiplying the collected image sensor data with a simple binary mask of different radial thicknesses. A spectrum was then constructed by summing these binned images across each wavelength the corresponding filter was set to. Further processing of the spectra used an iterative polynomial algorithm to remove the broad fluorescent background^49^.

### Fabrication of silica coated gold nanoparticles with dyes

All chemicals were purchased from Sigma Aldrich (St. Louis, USA). In short, a surfactant free and seed-mediated growth method was utilized to create stable GNS particles. A 12-nm seed solution was first prepared by adding 15 mL of 1% trisodium citrate to 100 mL of 1 mM HAuCl4 solution under vigorous stirring and boiling conditions. Upon the solution’s color change from black to red, the mixture is cooled and filtered through a 0.22-μm nitrocellulose membrane. A new solution consisting of 10 mL of deionized water, 10 µL 1 N HCl, and 493 μL HAuCl4 was then prepared and placed under moderate stirring. Addition of 100 μL of 12 nm seed particles, 50 μL of 2 mM AgNO3, and 50 μL of 0.1 M ascorbic acid produces 0.1 nM gold nanostars that are roughly 50 nm in size from tip to tip. Addition of more AgNO_3_ at increased pH levels afterwards creates silver coated gold nanostars, which further enhances the SERS enhancement. Adding 10 μL of 29% NH4OH and 50 μL of 0.1 M AgNO_3_ produces the coated particles and the solution color changes from blue to brownish red. The particles are subsequently mixed with 10 μL of 1 mM of SERS dye (DTTC, IR 780). The particles and dye are then enclosed together with the addition of Tetraethyl orthosilicate (TEOS). After a brief wash and resuspension in 1 mL deionized water, the silica coated particle dye combination is finally capped off with the addition of 100 μL of 1 mM 1 k polyethylene glycol (PEG) solution. Details in this synthesis are described in greater detail in a previous study^17^.

### Fabrication of gel phantom

All materials were purchased from Sigma Aldrich (St. Louis, USA). A 3% w/v agarose gel containing a concentration of 0.1 nM particle-dye is embedded on a small 5 cm diameter petri dish. Multilayered samples were fabricated by stacking the particle-dye embedded gels unto each other with a very thin layer of agarose gel solution to solidify the layers together. Quantum dots with a PbS core and emission peak wavelength at 900 nm was used in the gel lettering for demonstrating the XY imaging.

## Supplementary Information

Supplementary Figures.

## Data Availability

The datasets generated during and/or analyzed during the current study are available from the corresponding author on reasonable request.
