# Supplementary material for: Optical recognition of constructs using hyperspectral imaging and detection (ORCHID)
Source: Sci Rep. 2022 Dec 7;12:21141. doi: 10.1038/s41598-022-25735-9 (PMC9729193; doi:10.1038/s41598-022-25735-9)
Supplement: Supplementary file 1 — Supplementary Figures. [file 41598_2022_25735_MOESM1_ESM.docx]

**Supplementary Information**


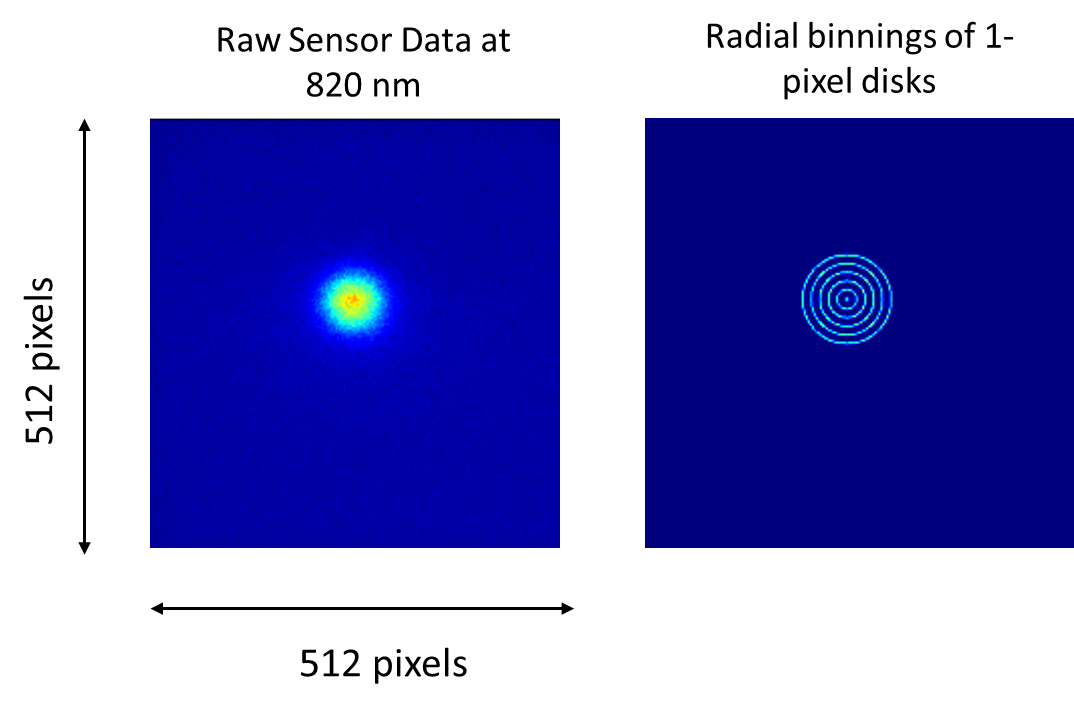


**Supplementary Fig 1:** Raw sensor output at captured at 820 nm and the binary radial mask used to selectively choose spatially offset pixels. The radial masks correspond to the offsets used in **Fig.** **4**.

**
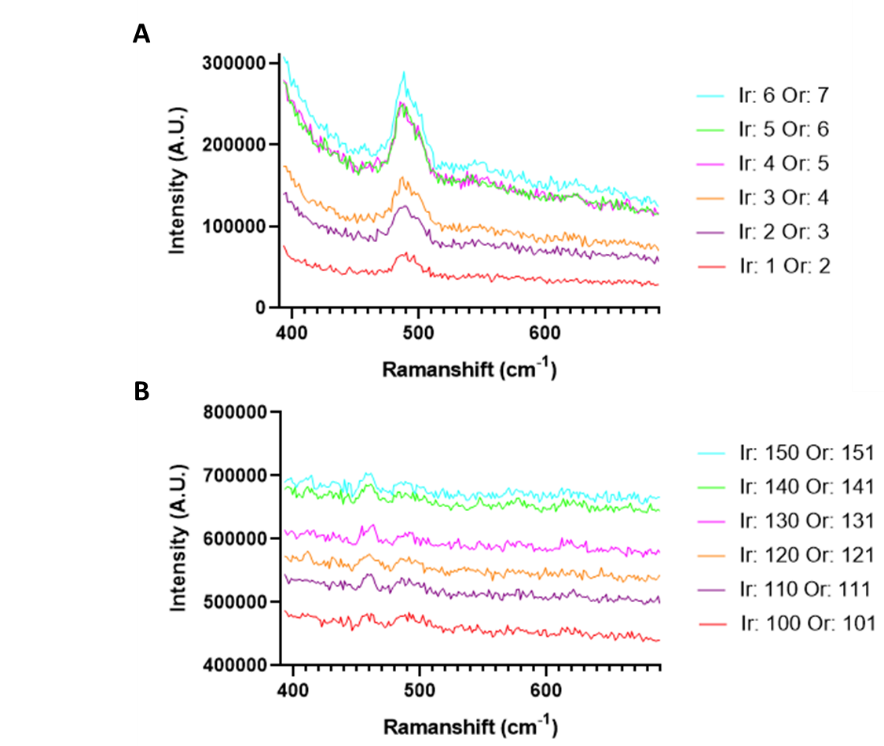
**

**Supplementary Fig. 2A:** Alternative spatial offset configurations with radial binning configurations for an embedded two-layer SERS gel phantom (See **Fig. 4**). Ir and Or stand for inner and outer radius of the binning disk used on the raw captured image. Note that the smaller configurations have a much lower signal to noise ratio as there are less pixels to collect light, leading to highly irregular spectral shapes. **Fig. 2B** shows thicker binning configurations which result in a smearing of signal across the layers as the binned pixels will now sample a larger Z slice of the sample.
